# Development of Surgical and Visualization Procedures to Analyze Vasculatures by Mouse Tail Edema Model

**DOI:** 10.1186/s12575-021-00159-3

**Published:** 2021-11-11

**Authors:** Shinji Kumegawa, Gen Yamada, Daiki Hashimoto, Tsuyoshi Hirashima, Mizuki Kajimoto, Kyoichi Isono, Kota Fujimoto, Kentaro Suzuki, Kazuhisa Uemura, Masatsugu Ema, Shinichi Asamura

**Affiliations:** 1 Department of Plastic and Reconstructive Surgery, Graduate School of Medicine, Medical University of Wakayama, Wakayama, Japan; 2 Department of Developmental Genetics, Institute of Advanced Medicine, Medical University of Wakayama, Wakayama, Japan; 3 Department of molecular Physiology, National Cerebral and Cardiovascular Center, Suita, Osaka Japan; 4 The Hakubi Center/Graduate School of Biostudies, Kyoto University, Kyoto, Japan; 5 Laboratory Animal Center, Wakayama Medical University, Wakayama, Japan; 6 Department of Stem Cells and Human Diseases Models, Research Center for Animal Life Science, Medical University of Shiga, Otsu, Shiga Japan

**Keywords:** Lymphedema, Lymphatic vasculature, Tail model, Live imaging, Transparent sheet, 3D reconstruction, Light sheet fluorescence microscopy(LSFM)

## Abstract

**Background:**

Because of the high frequency of chronic edema formation in the current “aged” society, analyses and detailed observation of post-surgical edema are getting more required. Post-surgical examination of the dynamic vasculature including L.V. (Lymphatic Vasculature) to monitor edema formation has not been efficiently performed. Hence, procedures for investigating such vasculature are essential. By inserting transparent sheet into the cutaneous layer of mouse tails as a novel surgery model (*the* ***T****ail* ***E****dema by* ***S****ilicone sheet mediated* ***T****ransparency protocol; TEST*), the novel procedures are introduced and analyzed by series of histological analyses including video-based L.V. observation and 3D histological reconstruction of vasculatures in mouse tails.

**Results:**

The dynamic generation of post-surgical main and fine (neo) L.V. connective structure during the edematous recovery process was visualized by series of studies with a novel surgery model. Snapshot images taken from live binocular image recording for *TEST* samples suggested the presence of main and elongating fine (neo) L.V. structure. After the ligation of L.V., the enlargement of main L.V. was confirmed. In the case of light sheet fluorescence microscopy (LSFM) observation, such L.V. connections were also suggested by using transparent 3D samples. Finally, the generation of neo blood vessels particularly in the region adjacent to the silicone sheet and the operated boundary region was suggested in 3D reconstruction images. However, direct detection of elongating fine (neo) L.V. was not suitable for analysis by such LSFM and 3D reconstruction procedures. Thus, such methods utilizing fixed tissues are appropriate for general observation for the operated region including of L.V.

**Conclusions:**

The current surgical procedures and analysis on the post-surgical status are the first case to observe vasculatures in vivo with a transparent sheet. Systematic analyses including the FITC-dextran mediated snap shot images observation suggest the elongation of fine (neo) lymphatic vasculature. Post-surgical analyses including LSFM and 3D histological structural reconstruction, are suitable to reveal the fixed structures of blood and lymphatic vessels formation.

**Supplementary Information:**

The online version contains supplementary material available at 10.1186/s12575-021-00159-3.

## Background

Onset of chronic edema in cancer and geriatrics is getting more reported these days.

Presence of the complex vascular network structures has delayed observation of L.V. and blood vessels. Such observation is particularly required to analyze the aberrant vascular structures in pathogenic conditions [1–3]. Among such pathogenesis, edema formation is noted as abnormal changes of L.V. (Lymphatic Vasculature). Accumulation of tissue fluids, which frequently includes various inflammatory products, is a prominent factor leading to form edema. Such accumulation can be caused by aberrant reduction of liquid drainage or augmented production of tissue fluids. It has been known that edema formation hampers the QOL of patients frequently leading to accelerated inflammatory reactions. Post-surgical edema formation in various malignant cancers including prostates and breasts is increasing in the modern “aged” society. Surgical removal of target tissues including prostatic and breast region often leads to the adjacent tissue damage and removals of lymphatic structures depending on their types on malignancy. After such procedures, edema is gradually formed due to the aberrant disorganization of L.V. in patients [4–6]. Thus, precise observation and analysis of the vascular structures are particularly essential to treat chronic edema.

In order to treat and cure edema in patients, analysis of the causative pathological incidents is essential by using experimental animal models. Generally, animal models have been developed to improve many analyses on vascular, developmental studies [7–9]. To proceed analysis for model animal studies, detailed observation of the dynamic vasculature in the target tissue areas is essential. Hence, analytical procedures of L.V. and blood vessels in post-operative surgeries in mouse models are becoming more important in vessel biology and surgical methodology fields.

Abnormal connections including neo-vasculature formation in L.V. have been suggested in edematous conditions. Observation of L.V. and blood vessels requires sophisticated visualization procedures. Previous works have been increasing in the medical biology of L.V. for pathogenic conditions and cancer [10–12]. To get breakthrough for the analysis of edematous condition, efficient live conditioned analytical procedure of the operated area is essential.

It has been generally speculated that fine (neo) L.V. has been generated particularly for the operated area which are expected to play roles for the gradual decrease of edematous conditions as recovery processes [13]. Visualization procedures through various dye staining has hitherto been applied to observe vasculatures associated with edema including L.V. and blood vessels. In order to get advantages in such visualization processes, a novel surgical procedure was developed as *the* ***T****ail* ***E****dema by* ***S****ilicone sheet mediated* ***T****ransparency protocol* (*TEST*) allowing L.V. observation. The method utilizes the transparent silicone sheet insertion above the blood vessels and the L.V. in the mouse tail to observe of the live vascular tissues in vivo. By utilizing the video through a binocular microscope, it is feasible to observe L.V. after its surgery**.** In order to evaluate the surgical procedures and several visualization processes, we also examined different types of visualization procedures post-surgically in the mouse tail edema model. In order to perform other visualization analysis of the L.V, light sheet fluorescence microscopy(LSFM)and 3D histological image reconstruction after processing the fixed tail tissue by transparent kit (CUBIC system) were utilized. We performed series of histological analysis using 3D reconstruction of vasculature structures in mouse tails. Introduction of the new *TEST* procedures with visualization techniques are discussed as novel strategies to analyze post edematous conditions which are expected to improve strategies to treat post-surgical edemas.

## Results

### Development of the Post-Surgical Tail Edema by the *TEST* Procedure

**Fig. 1:**
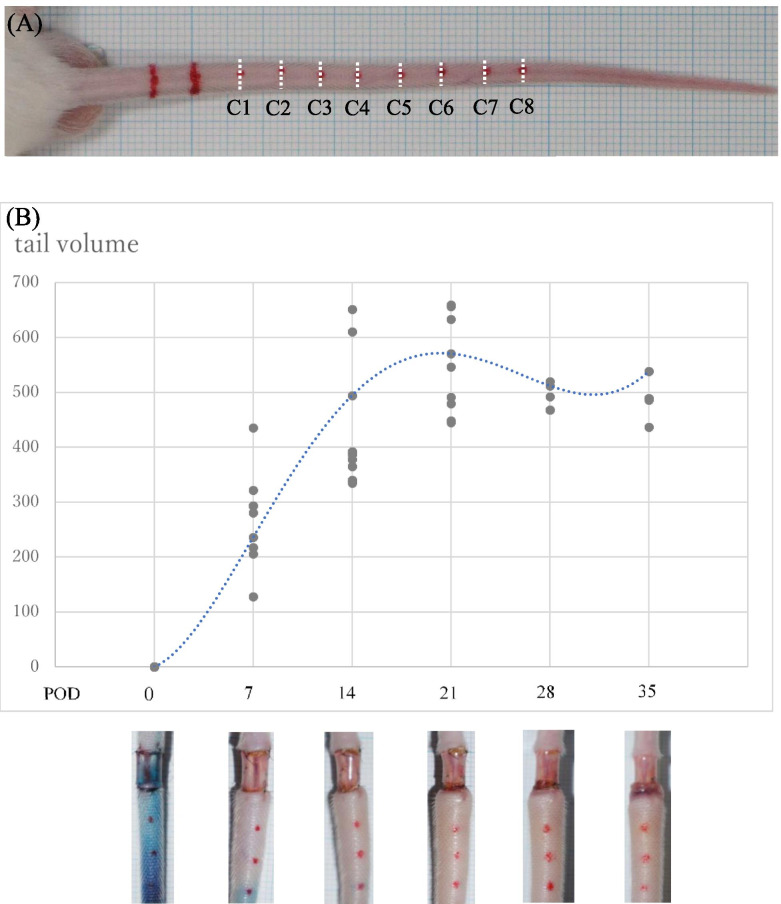
Parameters for post-operative tail volumes were shown by the calculations as previously reported (by using the parameters (C1, C2,…., C8) in Fig. 1**A**) [14]. Figure 1**B** shows the sequential observation of the swelling status of *TEST*-operated mouse tail with the illustration for the volume calculations. POD0 (post-operated day 0) and the gradual increase (POD0–21) and plateau (POD21–35) points of the operated tails were shown (Fig. 1**B**)

**Fig. 2:**
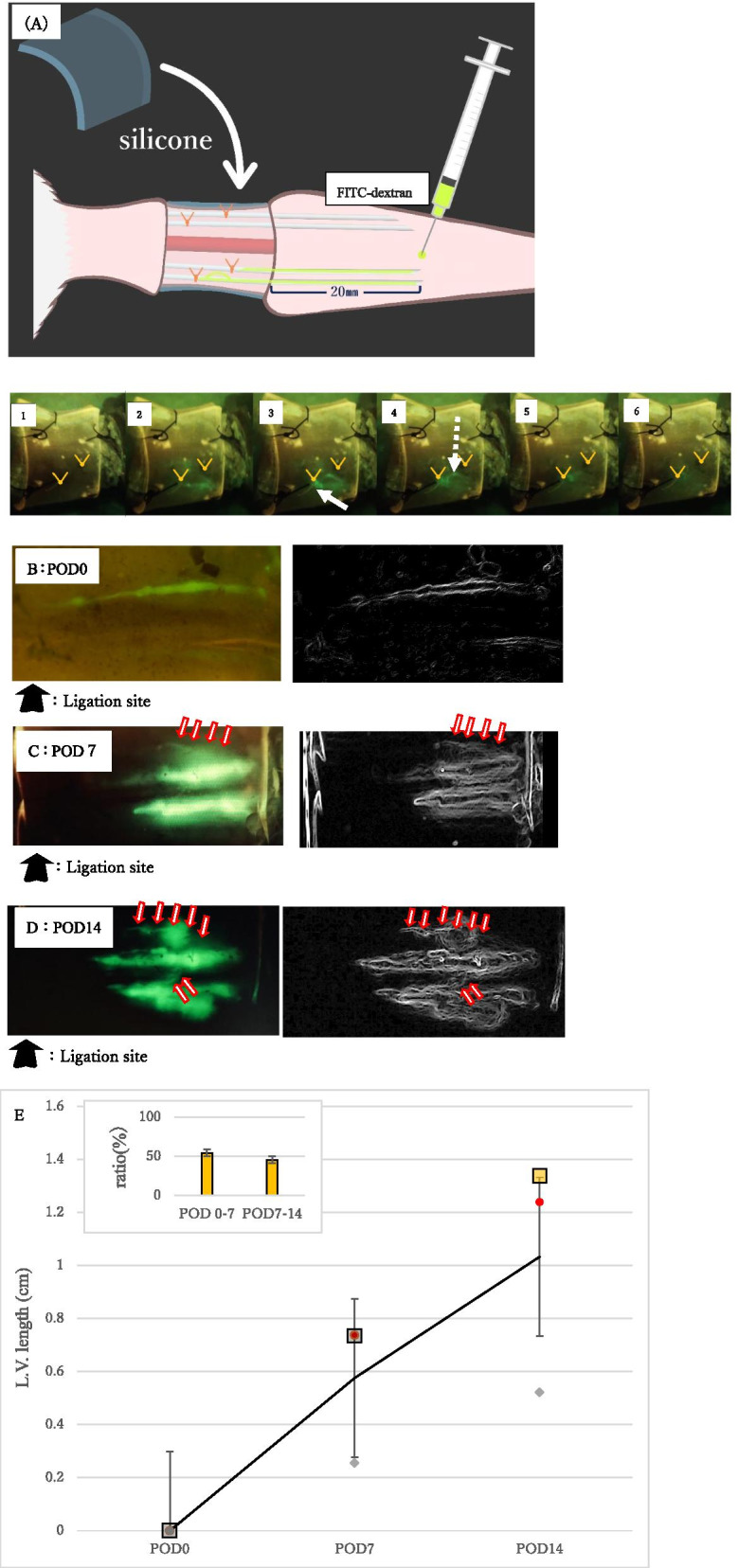
Fig. 2 shows images of a computer screen observed with a fluorescence microscope, taken shots at 60i 17 M (FH), 1920 × 1080 (60i), average bit rate 17Mbps and 30 frames per second, and snapshots were extracted by the movie. Dynamic L.V. formation in the operated area through transparent window was suggested in live conditions at POD21. (1)–(6) by the rapid distribution of the injected FITC-dextran (green). An experimental illustration shows the distribution of the FITC-dextran in the main L.V. and fine (neo) L.V. and the connections (**A**). In order to further demonstrate the efficacy of visualizing the elongating fine L.V. structure, plotting of the elongated branches was shown. Gradual increase of the post-surgical branched-fine L.V. elongation was plotted (**B**, **C**, **D** and **E**) during POD7–14. Rather stable degree of the its increase ratio was suggested (inset graph)

Practical procedures for the *TEST* model were developed in the current study. As shown in Fig. 1 and 2A, 0.5 mm thin transparent silicone sheet (Tigers Polymer, Osaka, Japan) was carefully inserted and sutured with 7–0 nylon to the operated site (5 mm square) in the mouse tail (See Methods). By the current experimental protocols, detailed post-surgical analysis of the vasculatures in the operated area is possible.

To examine the time course and conditions of post-surgical edema formation, sequential observation of the swelling status of treated tail was shown (Fig. 1B). In this procedure, it specifically ablates L.V. restoring the cutaneous layer of the operation site preventing side effects in post-surgical conditions by the sheet insertion.

Immediately after the surgical treatment of *TEST*, no prominent swelling was observed between POD0 (post-operated day 0) and POD3 (data not shown). As seen in the photos of treated tails, no abnormal bleeding nor inflammatory reactions were observed. To show such plateau status of the swelling, fixed tail location showing the gradual increase (POD0–21) and plateau (POD21–35) status were marked with dots (Fig. 1B). The calculation of the edematous tail volume was performed following the previous reports [14]. As observable by the graph of tail volumes, prominent swelling was maintained between POD21–35.

Additional wounding reaction and necrosis were rarely observed post-surgically. Compared with the previous simple surgical procedure to obtain edema formation (such as 10 days - 2 weeks of time) [15, 16], the current procedure enabled longer time of edema observation. Altogether, the current *TEST* procedure yielded reliable chronic edema formation post-surgically at higher frequency. Hence, the *TEST* can be utilized for chronic lymphatic edema formation rather than observing the acute phase of edema formation. Due to the obstruction of lymphatic structures at the operation site, analysis on the subsequent changes of adjacent lymphatic structure is the main concern (see below).

### Visual Inspection for the Operated Vasculatures

In the case of human clinics, either PB (Patent blue dye) or ICG (Indocyanine green dye) were utilized for the inspection for the operated site [17–20]. PB mediated visualization has been often utilized for evaluating various surgical procedures as it is stable for several manipulations and visible in optical wavelength light. Although application of PB is a feasible procedure, detection of fine (neo) vasculature system is often not possible due to its relatively low sensitivity by optical wave length-light. Hence, visualization and in vivo imaging by various dye injection is becoming frequently reported [21–24]. Injected PB after the current procedure showed the successful ligation and enlargement of the main L.V. (Additional file 3B, see Methods). To get advantages of the current transparent sheet application with fluorescent dye mediated visualization, FITC (Fluorescein isothiocyanate)-dextran (FD2000s, 60,842–46-8, SIGMA) was injected 2 cm peripherally from the operation site in subcutaneous dorsal aspect of tail (Fig. 2A). After confirming the plateau level of edema formation (Fig. 1, POD21–35), vascular analysis was performed to examine the elongation of neo-vascular formation related with the gradual recovery of edema. To observe such condition, the FITC-dextran was injected peripherally from the operation site and dynamic vasculature formation in the operated area through transparent window was examined in live conditions at POD21. Figure 2, (1)–(6) show the rapid distribution of the injected FITC-dextran; absence of the FITC-dextran signal before the injection (1), the subsequent rapid distribution of FITC-dextran along with the main L.V. after 0.1 s of injection (2), significant accumulation of FITC-dextran induced by the ligation site after 0.3 s of injection (white arrow, 3), appearance of the fine (neo) L.V. along with the main L.V. (shown by white dotted arrow after 0.5 s, 4), decreased FITC-dextran signal in the upper fine (neo) L.V. and reduced level of the FITC-dextran accumulation at main L.V. (5) and disappearance of the FITC-dextran signal in L.V. after 1 s (6). An experimental illustration shows the distribution of the FITC-dextran in the main L.V. and fine (neo) L.V. connections (Fig. 2A). The injection needle is placed adjacent to the L.V.s so that such subcutaneously injected FITC-dextran reaches to such L.V.s. Such observations suggest the development of fine (neo) L.V. formation adjacent to the L.V. structure (Lower part of Fig. 2A).

In order to further demonstrate the efficacy of visualizing the elongating fine structure, plotting of the elongated L.V. branches was shown. Detection of elongating fine (neo) L.V. by snapshot images was shown after *TEST* procedure. At the time of operation (POD0), elongation was not detected (Fig. 2B). Elongating fine (neo) L.V. was shown at POD7 (Fig. 2C, shown by red arrows, Right side figures are black and white images of the left figures). Further elongation of L.V. was shown by snapshot images after *TEST* procedure (Fig. 2D). Gradual increase of the post-surgical branched-fine L.V. elongation was shown (Fig. 2B-E) during POD7–14. Significant degree of its increase was suggested (The increase ratio of the branch- elongation is shown in the inset graph).

Rapid and dynamic distribution of the injected FITC-dextran suggested the post-surgically elomgating L.V. structure. Thus, the current results suggest the potential utility of rapid examination of the fine (neo) and the main L.V. structure during the recovery process of edema formation. After such vasculature elongation was detected, 3D analysis utilizing LSFM and 3D histological reconstruction was performed (see below).

### Visualization of the Transparent Post-Operative Tissues by Light Sheet Florescence Microscopy (LSFM)

**Fig. 3:**
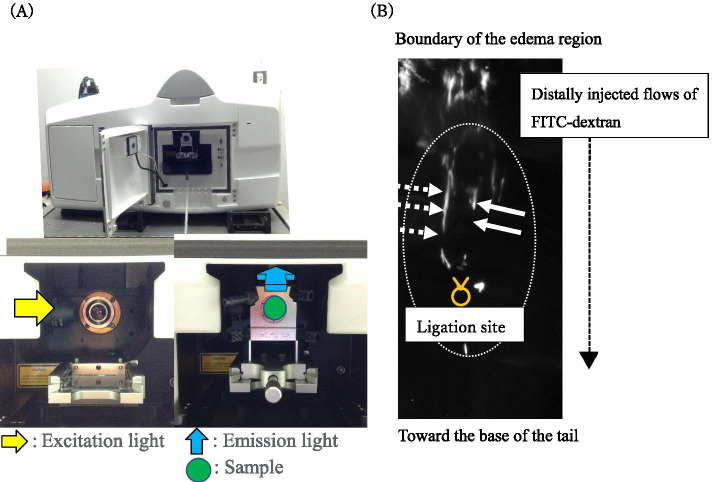
Fluoresce images were acquired with light sheet fluorescence microscopy (LSFM), (ZEISS Z.1, Germany). The LSFM system set up is shown (**A**). PFA fixed specimen was placed at sample fold (green circle) with Excitation light (yellow arrow) toward the detection device by Emission light (blue arrow). The distribution of FITC dextran signal was suggested the presence of main L.V. (shown by two white arrow) and presence of presumptive fine (neo) L.V. (shown by white dotted arrow in (**B**)). It was suggested that main and fine (neo) L.V. tended to extend to the ligation site (yellow mark, (**B**))

Procedures to generate tissue transparency are getting advanced these days [25–30]. To achieve precise observation of vasculatures by the fixed transparent operated regions, Clear, Unobstructed Brain/Body Imaging Cocktails (CUBIC) Protocol II mediated observation was performed [31, 32]. LSFM is generally useful to observe rather large transparent samples (Fig. 3 (A)) [27, 33]. After tissue transparency processing, FITC-dextran injection and subsequent microscopy observation is possible with LSFM.

In this study, we used a 2 mg/ml solution of tetramethylrhodamine-conjugated lysine fixable dextran of 2000 kDa (Invitrogen, Carlsbad, CA) to fix its distribution in the L.V. and observed it by LSFM [34]. Figure 3 (B) shows structures of L.V. shown by the transparent region. By such LSFM data, white line (candidate signal for main L.V. shown by white arrows) appeared to extend to the ligation site (yellow mark) (Fig. 3 (B)). In addition, elongated and curved fine (neo) L.V. was also suggested (candidate fine (neo) L.V. shown by white dotted arrows). Other LSFM picture also showed the presence of branched bud-like L.V. structure at POD14 (Additional file. 1). Taken together, the presence of new L.V. in the ligated areas of L.V. was suggested.

### 3D Histological Image Reconstruction Showing the Lymphatic Vasculatures and Blood Vessels by *the TEST* Protocol

Transparent sample analysis for visual observation is getting more reported in medical science [25, 27, 35–41].

**Fig. 4:**
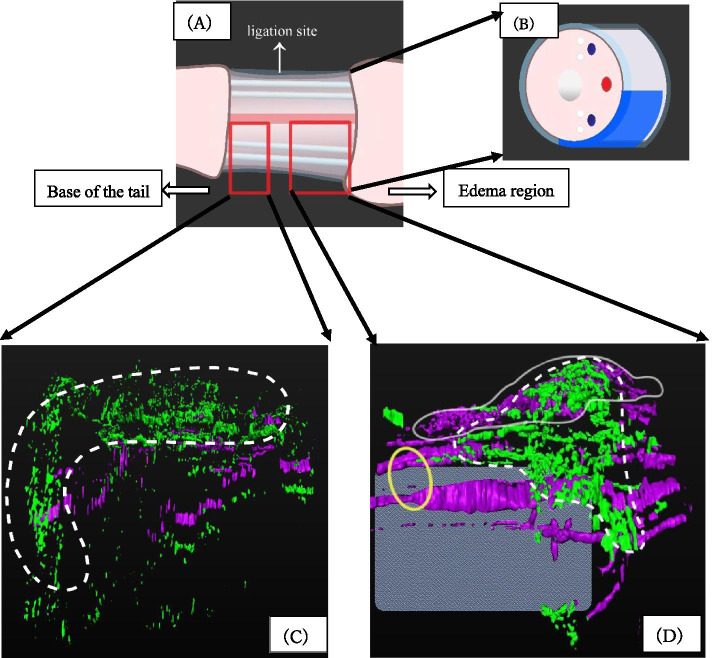
The area corresponding to the 3D image is indicated by the red square line (**A**). The main L.V.(white colored) runs along the sides of the tail and the lateral veins (blue colored) and its distribution is also shown (**B**). The ventral artery runs along the tail ((**B**): red colored). Total number of 533 serial sections of fluorescent stained (either CD31 or LYVE1) images were incorporated as digital data and processed by Amira software to construct 3D images of edema and its adjacent regions (**C**, **D**). In more proximal side from the operated region (adjacent to the base of the tail), generation of fine blood vessel was observed by green signal ((**C**): in dotted white circle region). As for the operated region close to the edema, the presence of two main enlarged L.V. by surgical ligation was clearly observed ((**D**); yellow circle region). Fine (neo) hyperplastic L.V. were observed by the purple signals ((**D**): white circle region), fine (neo) blood vessel formation was observed by the green signals ((**D**): dotted white circle region)

3D histological reconstruction technique is valuable to reveal the tissue structure [42–45]. In order to characterize the pathogenesis induced by the current surgical model, 3D reconstruction of post-surgical histological images was constructed. Total number of 533 serial section of fluorescent immunostaining images were incorporated as digital data and processed by Amira software to construct 3D images of edema and its adjacent operated regions (Fig. 4 (A)-(D)).

Cross-section of the *TEST* operated specimens (Fig. 4 (C), (D)).

The area corresponding to the 3D image is indicated by the red square line (Fig. 4 (A)). The main L.V.s (white colored) run along the sides of the tail and the lateral veins (blue colored) and its distribution is also shown (Fig. 4 (B)). The ventral artery runs along the tail (Fig. 4 (B): red colored).

By series of 3D movies (see Additional file 2), the next observations were obtained.

Toward the base of the tail in the operated region, generation of fine blood vessel was observed by green signal (Fig. 4 (C): dotted white circle region). Altogether, the prominently developed blood vessels at the peripheral edge of the silicone sheet, and underneath of it (Fig. 4 (C): dotted white circle region) were observed. In contrast, generation of new L.V. was not confirmed in such region (absence of prominent purple signals in dotted white circle). Due to the ligation of main L.V., it may be possible that new L.V. generation was not prominent.

In the operated region adjacent to the edema, the presence of two main enlarged L.V. by surgical ligation was clearly observed (Fig. 4 (D); the ligation site was shown by yellow circle). Associated with the *TEST*, fine hyperplasic L.V. were observed adjacent to the cutaneous epithelia next to the operated site and just below the silicone sheet (Fig. 4 (D): purple signal in the white circle region). Such L.V. located adjacent to blood vessels (Fig. 4 (D)). Fine blood vessels (Fig. 4 (D): green signal in dotted white circle region) were also prominent at the distal region of the silicone sheet. In deeper layer of such region, lymphogenesis was not prominently confirmed (Fig. 4 (D): absence of the fine purple signals shown in the grey box region). This indicates the prominent differences of post-surgical lymphatic responses between surface and deep layer of the operated region. Such differential responses of L.V. and blood vessels were suggested for the first time.

## Discussions

### Animal Models for Edema and Visualization Analysis

In human tumor therapy fields, formation of chronic edema hampers the QOL of patients who received removal of primary cancers [46–48]. Given the highly frequent onset of chronic edema in cancer and geriatrics, animal models of edema are getting more required. It has been reported that estimation and prediction for the onset of post-surgical edema is practically not feasible in many cases even after the careful surgical treatments [4, 49]. Examination of such chronic development of post-surgical edema is thus essential which ideally predicts and prevents its onset. Definition of acute and chronic of edema formation has been discussed in experimental animal models and in clinical patients [15, 50–52]. Diagnosis of chronic type of lymphedema is generally determined post-surgically after a few years etc. in patients [50]. Long term edema formation such as weeks of period, is generally referred as chronic types of animal models. Application of transparent sheets by surgical models has been performed to get advantages of its elasticity and stabilize the operated area and prevent its shrinkage [53, 54]. However, taking advantages of the transparency of the sheet to observe the underlying vessels has not been performed.

By the current *TEST* procedure, observation of dynamic state of vasculatures is useful to examine the post-operative status. Surgical models of acute and chronic phase of lymphatic edema formation of tail have been modified. In case of mouse hindlimb edema formation, radiation-induced phenotype is often described as chronic type of edema [55–57]. Besides hindlimbs, mouse tail has been often utilized to analyze edema by several surgical procedures. Compared with hindlimb models, tail region is anatomically relatively simple without well-developed muscular structures and its surgical treatment does not necessary lead to impair essential physiological status of mice in laboratory housing conditions. Table 1 summarizes experimental models for tail edema formation listing their experimental advantages and disadvantages. Simple surgical stripping models with ligation of L.V. generally showed outcomes with frequent infection and necrosis (A) [58] . Edema formation model has been originally reported by simple ablation of L.V. such as by electric knife mediated operation (B) [59]. Performing simple invasions for L.V. leads to not only L.V.-mediated fluid accumulation, but also damaging the operated site and underlying dermis [60]. Injuries of cutaneous layers often leads to shrinkable ulcer and abnormal keloid formation in the operated area. Thus, further modification of surgical model is required for specifically modulating vessels by utilizing sheets or collagens with ligating L.V. in the operated area (C) [16, 34, 53].

**Table 1:**
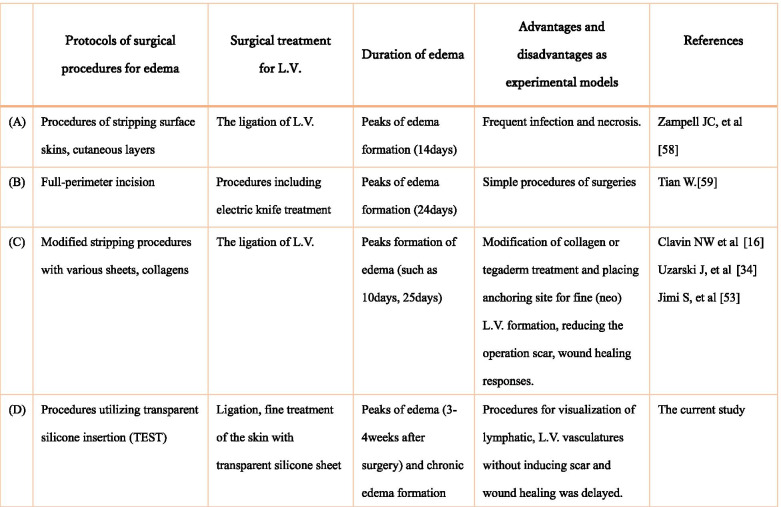
Summaries of the experimental models for tail edema formation. Historically, simple surgical stripping with ligation of lymphatic vesicle yielded not only experimental edema but frequent infection and necrosis (A). Ablation by electric knife mediated operation improved the efficiency of operation but often damaged the nearby tissues (B). Formation of edema by simple invasion of lymphatic vesicle frequently damaged operated site and the nearby dermis. Modification of the surgical procedures with various experimental non-transparent sheets or collagen have been performed (C). Transparent sheet insertion of the operated area allows visual inspection reducing the post-surgical side effects (D).

Generally, surface cutaneous epithelia and underlying layers including dermis with vasculatures are essential for cutaneous homeostatic regulation. Cutaneous surface modification by various grafting technics has been reported in tissue generation studies [53, 61, 62]. Relatively long period of observation for vasculogenesis in such graft tissues is expected to proceed tissue regeneration studies. Previous non-transparent sheet insertion surgeries only prevented the rapid onset of wounding and scar formation. In case of non-transparent silicone application, it was also utilized for surgical operation of stoma reconstructing its efficient functions [63, 64]. In that aspect, the current transparent sheet application is regarded as one of the first silicone-mediated procedures for visualization and improving stability of the operated area.

### Dynamic Status of Fine (Neo) – Main L.V. Formation in the Operated Site of *TEST*

Accurate ligation of L.V. and subsequent replacement by transparent silicone sheet (*TEST*) is reported in the current study. Insertion of transparent silicone allows observation of chronic edema formation. By this insertion, observation underneath of the silicone sheet in vivo becomes possible using fluorescent dye injection. Surgical ligation was confirmed by the PB injection staining (Additional file 3A, B). Rapid fluorescent staining and the snapshot observation from the main and fine L.V. suggested the presence of dynamic post-surgical L.V. formation (white dotted arrow in Fig. 2: (1)–(6)). Furthermore, examination for the extent of such fine L.V. elongation indicated the effective visualization of the transparent sheet mediated lymphogenesis. It is still speculative whether such newly developed connections were initiated adjacent from the main L.V. ligation site or not. Due to such ligation, increased volume and pressure were assumed as generated not only in the ligation site but along with the main L.V. It is thus possible that interconnection of such main - fine (neo) L.V. was dynamically generated close to ligation site. In case of human cancer clinics, after resection of primally tumor such as at breast/ prostate, careful ligation of several lymphatic nodes and vasculature was extensively performed [65–67]. Attention has been focused on the completeness of ligation or electric knife treatments as an efficient surgical procedure, but less attention has been focused on its subsequent effects in the adjacent regions. Hence, detailed analysis for the effect of ligation adjacent to the operated site is essential and the current analysis is one of the initial works showing dynamic changes of vasculature.

Treatments including post-surgical reconstructive surgery, self-drainages etc. may generally augment the generation of main – fine (neo) L.V. connections possibly reducing the extent of post-surgical edema. Hence, the current *TEST* procedure can be combined for various post-surgical analysis including various treatments. Connections between pre-existent L.V. and fine (neo) L.V. structures need to be analyzed also with L.V. generating activity [68]. IL6 (Interleukin6) has been one of the major factors regulating inflammation and also lymphogenesis [69]. Various factors affecting lymphogenesis such as IL6 can be applied in this *TEST* procedure related experiments [69, 70]. The finding of post-surgical connections of main – fine (neo) L.V. may open a research field for the post-surgical edema. Further analysis for the effect of surgical ligation can be studied by utilizing live vasculature images plus observation by detailed 3D analysis.

As for other post-operative visualization, comparisons for transparent analysis for LSFM observation was shown in the current study. LSFM is capable of imaging samples spanning orders of magnitude in space and time and it is suitable for observing transparent samples because of its optical depth focus and scanning characters [33]. Because general optical light depth by LSFM is around 6-8 mm, it can cover various fine (neo) L.V. around the ligation site in this experimental set up. Both LSFM and 3D imaging method possesses advantages and disadvantages. In the case of 3D histological reconstruction, long process of histological section preparation, staining and subsequent reconstruction are necessary. After performing such process, information of L.V. and blood vessels can be analyzed with CD31 (endothelial marker) and LYVE1 (L.V. endothelial marker) staining data. In the case of LSFM observation, tissue processing including transparent tissue preparation is still necessary. However, taking images after injection of FITC-dextran is sensitive without procedures of sectioning. Utilizing lysine conjugated FITC-dextran is essential, because of its retention inside the L.V. after fixation. Such fixable fluorescent dye enables to show the relatively longer fluorescent dye distribution between main- fine (neo) L.V. For both LSFM and 3D reconstruction analysis, continuous observation of the elongation proses of L.V. is not possible because of the fixation for observing sample. Because of the significant experimental time required for experiments and inability of continuous observation after fixation, the current type of visual binocular snapshot analysis through transparent sheet by *TEST* procedure will be particularly useful as the first experimental protocol.

## Conclusion

The new surgical procedures and analysis on the post-surgical edematous status of mouse models are introduced in the current study. Formation of tail edema by transparent sheet insertion was analyzed (*TEST* surgical procedure). Live images taken after the procedure suggested the presence of main and elongating fine (neo) L.V. formation for the first time. In addition to such live imaging, two histological observation experiments were performed.

Light sheet fluorescence microscopy (LSFM) mediated observation indicated the presence of fine L.V. formation. 3D histological image reconstruction also showed the fine structure of blood and lymphatic vessels adjacent to the edema region and in the surface cutaneous region. However, such two procedures can be performed only by fixation of post-surgical samples thus, not suitable for imaging for the L.V. elongation in vivo.

## Methods

### A Novel Lymphedema Mouse Tail Model (*TEST*)

We developed the mouse tail surgery of lymphedema as a new procedure (*TEST*) in this study. Anesthesia was performed by administering three types of mixed general anesthesia (Medetomidine(0.3 mg/kg), Midazolam(4 mg/kg), Butorphanol(5 mg/kg)) into abdominal cavity of mice. In the model, a 5 mm wide circumferential full-thickness skin section was excised 2 cm distal to the tail base to remove superficial L.V. The procedures were designed so that the vessels were clearly visible underneath the transparent silicone sheet (Additional file 3A). Improper visualization was judged by compressing blood vessels with fine forceps to avoid the central region of the surgical field was stagnant or the operated blood vessel was visible only in part of the transparent window region. A yellow arrow shows smooth distribution of endogenous blood along with the L.V. after the *TEST* surgery (Additional file 3A).

After patent blue (PB) was administered subcutaneously from the distal part of the tail to identify the position of deep L.V.s, they were extensively ligated with 10–0 nylon suture. After performing incision of the operated area of skin, examination of the location of L.V. and vein underneath skin and subsequent peeling off the L.V. was performed without inducing bleeding. Next, an examination was performed so that the injected PB did not show proximal leakage adjacent to the ligation and the L.V.s were expanded at the operation site (Additional file 3B: white dotted circles). A 5 mm wide rectangular silicon splint was fabricated from a 0.5 mm thin silicon sheet (125–18–11-01, Tigers Polymer, Osaka, Japan). Such silicone materials were selected by its transparency and flexibility as an insert membrane. The transparent silicone sheet was originally utilized for coverage of PC keyboard to secure transparent operation with flexibility and strength.

The transparent silicone sheet should clarify the next conditions of *TEST* surgical operation.

1. Flexible and solid as surgical material
2. Stable and durability as material for surgical operations including suture
3. Fitness and adhesiveness to the operation area, the curved structure of mouse tail

The silicone splint was sutured to the skin at both edges of the skin-peeled surgical field with 7–0 nylon, and the surgical procedure was completed. The silicone was ligated from the 0 o’clock direction (dorsal side of the tail) to avoid the blood vessels. To avoid excessive pressure on the blood vessels, the length of the silicone was finally adjusted ligating to the tail.

### Calculation of the Edema Status

The kinetics of edema formation after post-surgical *TEST* procedure were shown (Fig. 1). The degree of edema formation was calculated weekly using the truncated cone formula: V = l/4π (C1C2 + C2C3… + C7C8) [14]. Tail diameters were measured using digital calipers every 0.5 cm starting from the surgical site going distally toward the tip of the tail (Fig. 1A). The time point of post-operative day was indicated as POD including POD0 as the time of the operation. Each point in the graph represents increased tail volume compared with POD0 for each operated specimen (Fig. 1B). During POD0–21, gradual increase of edema formation was revealed by the tail volume and inspection of the corresponding swelling status (lower part of Fig. 1B). During POD 21–35, the plateau state of such edema formation was observed.

### Animals

Male ICR mice (2 months old) were purchased from CLEA Japan. All procedures and protocols have been approved by Animal Research Committee of Wakayama Medical University (approval number: 994). We have confirmed that all methods were carried out in accordance with relevant guidelines and regulations.

### Dynamic Video Recording of Binocular Microscope Images after FITC-Dextran Injection

In order to take sequential snapshot images of FITC-dextran (2000 kDa, 60,842–46-8, SIGMA) injected L.V. images, FITC-dextran was injected subcutaneously into 2 cm peripherally from the operation site by administering three types of mixed general anesthesia (Medetomi-dine, Midazolam, Butorphanol) into abdominal cavity (ICR mice; *N* = 9). Immediately after the injection (approximately till 5 s), video images were taken by binocular fluorescent microscope (Leica: M165 FC). Snapshot FITC images were shown at the interval of 0.5 s immediately after injection. Original video images were recorded by the form of MTS files and snapshots were extracted by the movie.

In addition to the tail volume measurement at each post-surgical points, increase of several stages of tail L.V. length was calculated between POD0–7 and POD7–14 (Fig. 2B-E). The increase ratio (shown as POD0–7 value by POD0–7/ POD0–14) was shown by the inset graph.

### CUBIC Protocol for Processing the Fixed Tail Tissue for Transparency

The entire tails were fixed overnight in 4% paraformaldehyde (PFA) in Phosphate- buffered saline (PBS). The fixed tails were harvested 5 mm centrally and 10 mm peripherally from the surgical site and then washed with PBS overnight at room temperature (about 25 °C). The samples were immersed in a 1:1 mixture of CUBIC-L solution (T3740, Tokyo Kasei Kogyo) and PBS for 1 day at 37 °C with gentle shaking and CUBIC-L solution at 37 °C for 2 days with shaking. They were washed with PBS overnight at room temperature. The samples were subsequently immersed in a 1:1 mixture of CUBIC-R solution (T3741, Tokyo Chemical Industry) and PBS with gentle shaking at room temperature for 1 day. The samples were then immersed in CUBIC-R with gentle shaking at room temperature for 2 days.

### Light Sheet Fluorescence Microscopy (LSFM) Observation of the L.V. in the Operated Area

Fluorescence images were acquired with LSFM (ZEISS Z.1, Germany) and the excitation laser (561 nm) corresponding to FITC-dextran was selected. Samples were immersed in a 1:1 mixture of silicone oil TSF4300 (Momentive Performance Materials, RI = 1.498) and mineral oil (Sigma-Aldrich, RI = 1.467) during image acquisition [31]. We prepared a customized sample holder for capturing the image of operated area. A fluorinated ethylene-propylene (FEP) tube with a refractive index almost equal to that of water was used to support the sample. The method is to insert rubber into the FEP tube to increase the contact area with the sample. The sample was held in place by applying alonalpha (TOAGOSEI CO, Japan) to the rubber surface. In order to perform the LSFM imaging, the PFA-fixed specimen was placed at the tissue folder (Fig. 3 (A) shown by green). Excitation light was illuminated (Fig. 3 (A) shown by yellow arrow) and emission light was captured by the sCMOS sensor (Fig. 3 (A) shown by blue arrow, Tokyo Instruments, Inc).

### Tissue Processing, Immunostaining and 3D Reconstruction Images

Tissue samples were fixed in 4% (wt/vol) PFA overnight, dehydrated in methanol (25–100%) washes and embedded in paraffin. The paraffin blocks were sectioned serially (10 μm thick) with a microtome (902,100, HM325, PHC, Japan), placed on slides, deparaffinized and rehydrated. Antigen retrieval was performed with either by citrate buffer or by autoclaving at 121 **°**C, 1 min. For primary antibody staining, the following antibodies were utilized: anti-CD31 (endothelial markers; 1/200, AF3628, goat, R&D systems) and anti-LYVE1 (L.V. endothelial marker; 1/100, ab14917, rabbit, Abcam). For secondary antibody reaction, the following antibody was utilized: Invitrogen Donkey anti-goat IgG Alexa Fluor 488 (1/200, Thermo Fisher Scientific). Total number of 533 serial section of fluorescent immunostaining images were incorporated as digital data and processed by Amira software to construct 3D images of edema and its adjacent regions (Fig. 4).

## Supplementary Information

**Additional file 1.** Additional file 1 shows another LSFM captured image of bud-like L.V. structure (shown by red circle). Such bud structure was detected at POD14. The ligation suture was remained as a black signal. The main L.V. locates left to the red circle in the picture.

**Additional file 2.** Additional file 2 2A, 2B In the lower side of such images, 3D reconstructed movie was attached corresponding to Y axis of reconstructed images. Additional file 2A is a 3D movie of Fig. 4(C) Y-axis. The movie shows the prominently developed blood vessels at the peripheral edge of the silicone sheet, and underneath of it. In contrast, generation of fine (neo) L.V. was not confirmed in such region. Additional file 2B is a 3D movie of Fig. 4(D) turning by Y-axis. The movie shows fine (neo) hyperplastic L.V. observed adjacent to the cutaneous epithelia next to the operated site and below the silicone. Fine (neo) blood vessels were prominent at the distal edge of the silicone sheet. In deeper layer of such region, lymphogenesis was not prominently confirmed.

**Additional file 3.** Additional file 3A, 3B Injected PB (Patent Blue dye) after the current procedure showed the successful ligation and enlargement of the main L.V. FITC (Fluorescein isothiocyanate)-dextran (FD2000s, 60,842–46-8, SIGMA) was injected subcutaneously 2 cm peripherally from the operation site (3A). 4A shows normal and undisturbed distribution of endogenous blood in the operated area (shown by yellow arrow). 3B shows the prominent enlargement of two main L.V.s due to the ligation (two white dotted circle area. Ligation was shown by the black suture).

## Data Availability

Not applicable (All data are included in the MS).

## Abbreviations

L.V.: Lymphatic vasculature
TEST: The tail edema by silicone sheet mediated transparency protocol
LSFM: Light sheet fluorescence microscopy
POD: Post-operated day
PB: Patent blue dye
ICG: Indocyanine green dye
FITC: Fluorescein isothiocyanate
CUBIC: Clear, unobstructed brain/body imaging cocktails
FEP: Fluorinated ethylene-propylene
