# Supplementary figures and images for: Development of Surgical and Visualization Procedures to Analyze Vasculatures by Mouse Tail Edema Model
Source: Biol Proced Online. 2021 Nov 11;23:21. doi: 10.1186/s12575-021-00159-3 (PMC8582144; doi:10.1186/s12575-021-00159-3)

**Additional file 1**

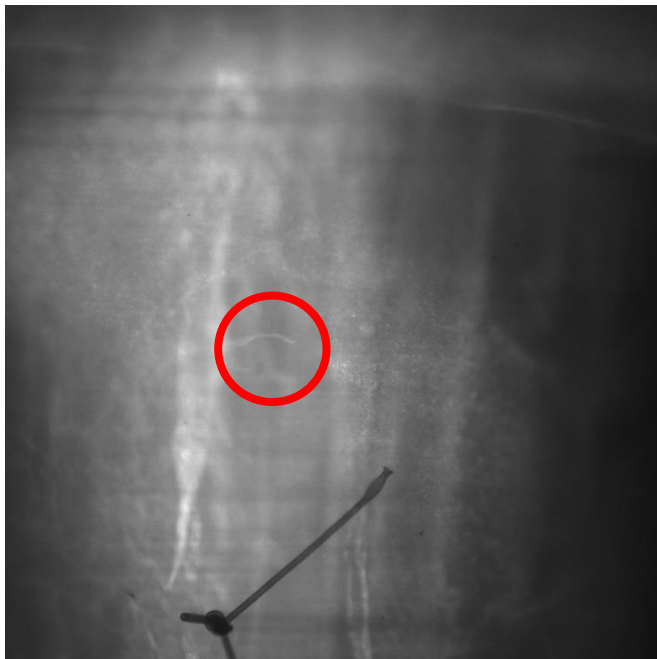

Supplement: Supplementary file 1 — Additional file 1. Additional file 1 shows another LSFM captured image of bud-like L.V. structure (shown by red circle). Such bud structure was detected at POD14. The ligation suture was remained as a black signal. The main L.V. locates left to the red circle in the picture. [file 12575_2021_159_MOESM1_ESM.pdf]

## Slide 1
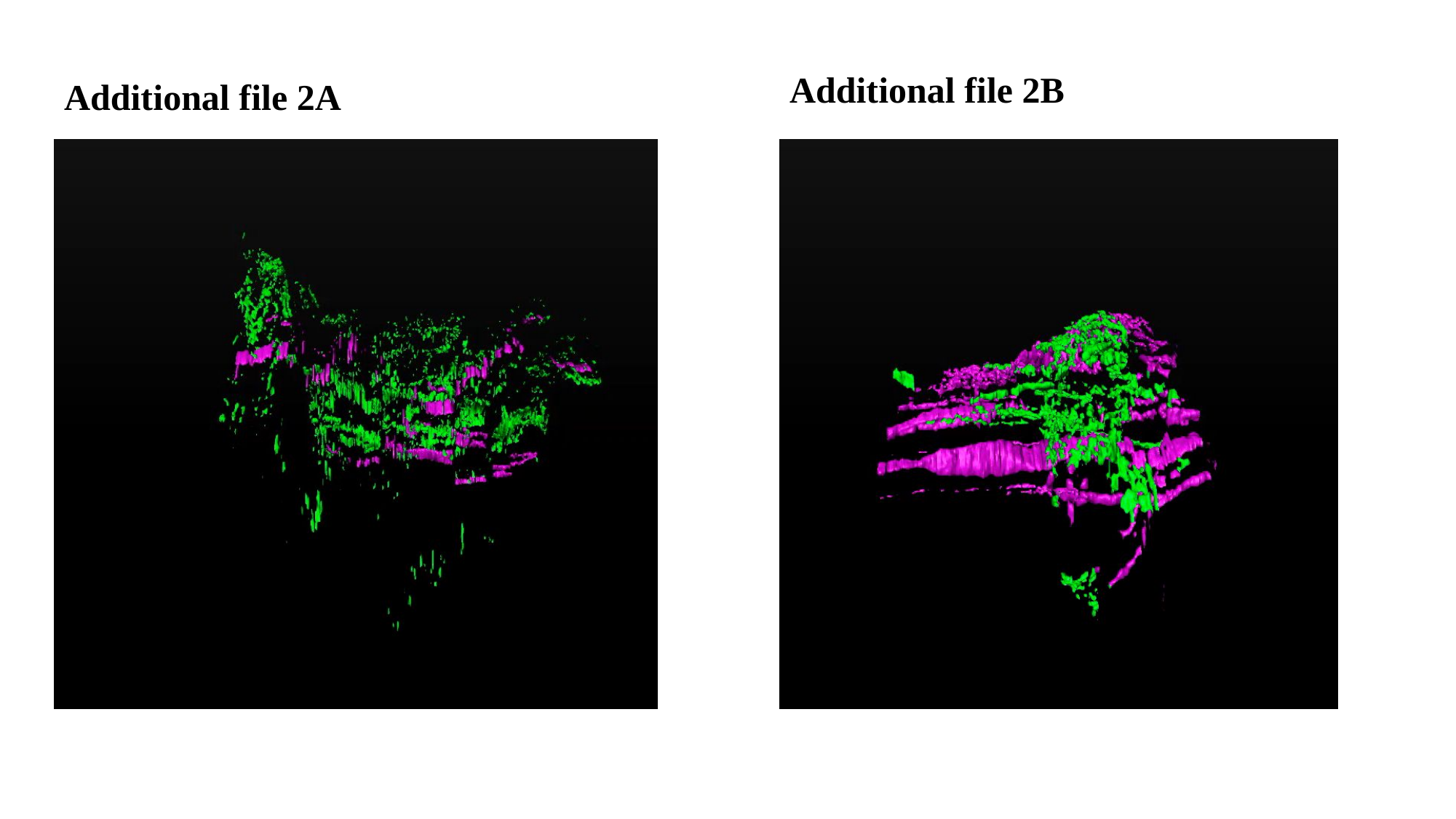

Additional file 2B
Additional file 2A

Supplement: Supplementary file 2 — Additional file 2. Additional file 2 2A, 2B In the lower side of such images, 3D reconstructed movie was attached corresponding to Y axis of reconstructed images. Additional file 2A is a 3D movie of Fig. 4(C) Y-axis. The movie shows the prominently developed blood vessels at the peripheral edge of the silicone sheet, and underneath of it. In contrast, generation of fine (neo) L.V. was not confirmed in such region. Additional file 2B is a 3D movie of Fig. 4(D) turning by Y-axis. The movie shows fine (neo) hyperplastic L.V. observed adjacent to the cutaneous epithelia next to the operated site and below the silicone. Fine (neo) blood vessels were prominent at the distal edge of the silicone sheet. In deeper layer of such region, lymphogenesis was not prominently confirmed. [file 12575_2021_159_MOESM2_ESM.pptx]

**Additional file 3A**

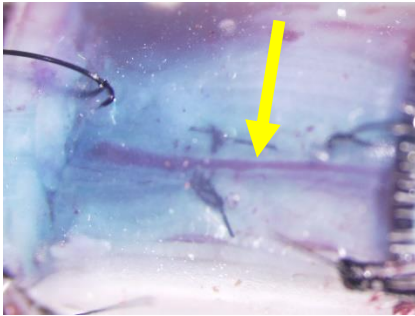

**Additional file 3B**

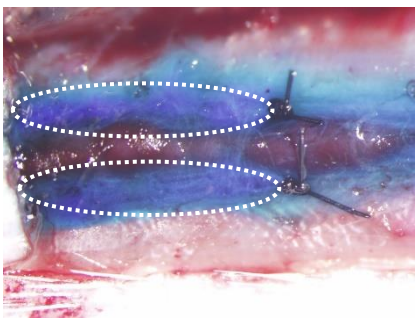

Supplement: Supplementary file 3 — Additional file 3. Additional file 3A, 3B Injected PB (Patent Blue dye) after the current procedure showed the successful ligation and enlargement of the main L.V. FITC (Fluorescein isothiocyanate)-dextran (FD2000s, 60,842–46-8, SIGMA) was injected subcutaneously 2 cm peripherally from the operation site (3A). 4A shows normal and undisturbed distribution of endogenous blood in the operated area (shown by yellow arrow). 3B shows the prominent enlargement of two main L.V.s due to the ligation (two white dotted circle area. Ligation was shown by the black suture). [file 12575_2021_159_MOESM3_ESM.pdf]
